# Influence of Solvents and Adsorption of Organic Molecules on the Properties of CVD Synthesized 2D MoS_2_

**DOI:** 10.3390/nano13142115

**Published:** 2023-07-20

**Authors:** Antun Lovro Brkić, Antonio Supina, Davor Čapeta, Lucija Dončević, Lucija Ptiček, Šimun Mandić, Livio Racané, Ida Delač

**Affiliations:** 1 Center for Advanced Laser Techniques, Institute of Physics, Bijenička Cesta 46, 10000 Zagreb, Croatia; albrkic@ifs.hr (A.L.B.);; 2 Physics Department, Faculty of Mathematics and Physics, University of Ljubljana, Jadranska 19, SI-1000 Ljubljana, Slovenia; 3 Department Physics, Mechanics and Electrical Engineering, Montanuniversität Leoben, 8700 Leoben, Austria; 4 Division of Molecular Medicine, Ruđer Bošković Institute, Bijenička 54, 10000 Zagreb, Croatia; 5 University of Zagreb, Faculty of Textile Technology, Prilaz baruna Filipovića 28a, 10000 Zagreb, Croatia

**Keywords:** 2D materials, MoS2, organic molecules, drop-casting

## Abstract

We present a simple method for modification of 2D materials by drop-casting of the organic molecule in solution on the 2D material under ambient conditions. Specifically, we investigated the adsorption of 6-(4,5-Dihydro-1*H*-imidazol-3-ium-2-yl)-2-(naphthalene-2-yl)benzothiazole methanesulfonate (L63MS) organic molecule on 2D MoS${}_{2}$. To better understand the effect of the organic molecule on the 2D material, we also investigated the impact of solvents alone on the materials’ properties. The MoS${}_{2}$ samples were synthesized using ambient pressure chemical vapor deposition. Atomic force microscopy, Raman spectroscopy, photoluminescence spectroscopy and optical microscopy were used to characterize the samples. The measurements were performed after synthesis, after the drop-casting of solvents and after the drop-casting of organic molecule solutions. Our results indicate that the used organic molecule effectively adsorbs on and prompts discernible changes in the (opto)electronic properties of the 2D material. These changes encompass variations in the Raman spectra shape, alterations in the photoluminescence (PL) signal characteristics and modifications in excitonic properties. Such alterations can be linked to various phenomena including doping, bandgap modifications, introduction or healing of defects and that the solvent plays a crucial role in the process. Our study provides insights into the modification of 2D materials under ambient conditions and highlights the importance of solvent selection in the process.

## 1. Introduction

Two-dimensional (2D) materials, such as graphene, transition metal dichalcogenides (TMDCs), hexagonal boron nitride (hBN), black phosphorous (BP), MXenes and 2D oxides, have experienced a surge in popularity since the discovery of mechanically exfoliated graphene using scotch tape [1]. This has prompted extensive research into their synthesis [2,3,4,5,6] and unique properties, which include atomic thinness, dangling-bond-free surface and high crystallinity [7,8,9,10]. Among others, they often exhibit promising (opto)electronic properties which can further be tuned, e.g., via external electronic modulation or surface adsorption, resulting in potential applications in numerous fields, such as electronics, photonics, catalysis and energy storage [1,11,12,13,14,15,16,17]. The broader applications of 2D materials have been hampered by the limitations inherent to their atomic-scale thinness and large surface area, which results in a high sensitivity to defects. For instance, procedures such as exfoliation or dispersion [18,19,20] required for the majority of chemical modifications could introduce numerous defects and it is known that a lot of them, such as BP, degrade over time in ambient environments [21,22]. Nonetheless, the large surface area of 2D materials presents opportunities for modifications [23,24,25,26,27,28,29,30,31,32,33,34,35], which could provide a means of mitigating these limitations and improving their properties. In particular, modification using adsorption of organic molecules with different geometries and/or functional groups could influence the properties of the 2D substrate layer in a controllable way.

This paper delves into the investigation of 2D molybdenum disulfide (MoS${}_{2}$), a member of the TMDC family and the potential avenues for its modification with organic molecules. Monolayer MoS${}_{2}$ is highly stable direct band gap semiconductor (crossing over from indirect band gap semiconductor in bulk and few layers due to quantum confinement) [36,37], whose band gap and optical properties are highly susceptible to tuning by physical methods like strain, temperature, doping or physisorption [38]. In addition to other outstanding properties such as high charge carrier mobility at room temperature [39], ability to withstand strains up to 11% [40] and piezoelectricity [41], it holds promise for prospective applications in fields like optoelectronics, nanophotonics and sensing. The wide applicability is further evidenced by the successful demonstration of several prototype devices, including but not limited to sensors, light emitters, photodiodes and phototransistors [38].

Here, we introduce a novel method for modifying MoS${}_{2}$ under ambient pressure by drop-casting a solution containing the organic molecule 6-(4,5-Dihydro-1*H*-imidazol-3-ium-2-yl)-2-(naphthalene-2-yl)benzothiazole methanesulfonate (L63MS) onto MoS${}_{2}$’s surface. This modification occurs at room temperature as the solvent evaporates. To comprehend this modification process fully, it became vital to explore the role of solvents. We scrutinized the influence of solvents on the properties of MoS${}_{2}$ prepared via chemical vapor deposition (CVD) [6], aiming to differentiate between non-functionalized, solvent-treated and solution-treated MoS${}_{2}$ samples.

Our study’s primary goal is to examine the potential impact of solvents on the properties of MoS${}_{2}$, independently of modification, as this could significantly influence its quality. A specific interest lies in the effects on 2D materials at a scale of a few micrometers or larger, i.e., on the morphology and structural integrity of single MoS${}_{2}$ flakes and the sample as a whole. Simultaneously, we’re exploring solvents’ roles in the modification procedure, studying their impact on both mechanical and optical properties of the MoS${}_{2}$ monolayer. By offering a detailed study on MoS${}_{2}$ modification and solvent impacts, we aspire to enrich the growing body of knowledge on modifying 2D materials under ambient conditions. This research may pave the way for developing new functional materials with enhanced characteristics and broad applications.

## 2. Materials and Methods

To investigate the modification of MoS${}_{2}$ using L63MS organic molecule and the effect of solvents on the material’s properties, we employed several techniques to measure the sample characteristics. Atomic force microscopy was used to obtain high-resolution topography images and measure the sample roughness. Raman spectroscopy was used to determine the material’s structural and vibrational properties. Photoluminescence spectroscopy (PL) was used to assess the material’s optical properties and optical microscopy was used to visualize and roughly assess the quality of the sample’s surface. Below, we provide a detailed description of our sample preparation, measurement and data analysis procedures.

### 2.1. 2D Material and Organic Molecule Synthesis

For the purpose of this research, MoS${}_{2}$ was synthesized on a SiO${}_{2}$ substrate using the chemical vapor deposition (CVD) technique. This resulted in distinctive monolayer MoS${}_{2}$ triangles with sizes ranging from 10 to 150 μm. For more details regarding the synthesis process, including the specific conditions and subsequent quality analysis of the MoS${}_{2}$ monolayers, please refer to our previous work [6].

**Figure 1:**
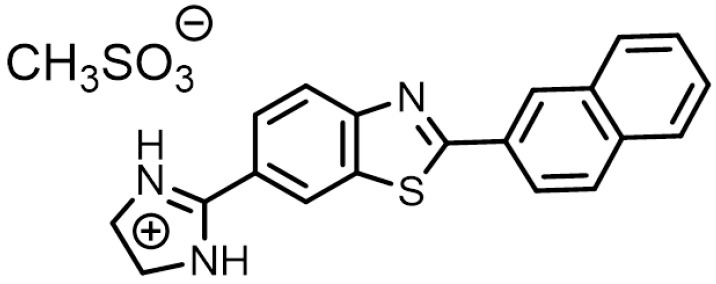
Bond-line formula of L63MS.

The organic molecule used in this study was synthesized using a well-established protocol [42,43,44] (Figure 1). The Pinner reaction was used to convert isomeric cyano-substituted 2-aminophenols into amidine derivatives, which then underwent a condensation reaction with aryl carboxylic acids. In this paper, the molecule will be referred to by its abbreviated name, L63MS.

### 2.2. Sample Preparation and Measurement

All measurements were performed using freshly prepared solutions and 2D materials within 12 h of preparation. The solutions were kept under refrigerated conditions (3 °C) and the 2D materials were kept under mild vacuum (100 mbar). The final characterization was preformed using the following:

AFM: The topography and morphology of the samples were characterized using a commercial AFM system NanoWizard 4 AFM Ultra speed manufactured by JPK (Berlin, Germany). The measurements were performed in tapping mode using TAP300Al-G probes obtained from Budget Sensors (Sofia, Bulgaria) with 10 nm radius of curvature, nominal spring constant of 40 N/m and a nominal resonant frequency of 300 kHz, at a scan rate of 1 Hz.

Raman and PL spectroscopy: The Raman and PL spectra of the samples were measured using a commercial Raman microscope (Qontor from Renishaw) equipped with a 532 nm laser.

Optical microscopy: The optical images of the samples were acquired using the metallurgical microscope DM2700 from Leica. The images were captured under white light illumination.

Data analysis: The AFM images were analyzed using JPK Data Processing software, version 6.1.198, JPK Instruments (Berlin, Germany) [45]. Raman and PL data were fitted, analyzed and visualized using custom Python code.

The AFM images were used to determine the thickness and surface roughness of the samples, while the Raman spectra were used to analyze the vibrational modes and defects in the MoS${}_{2}$ lattice. The PL spectra were used to study the electronic and optical properties of the samples.

**Figure 2:**
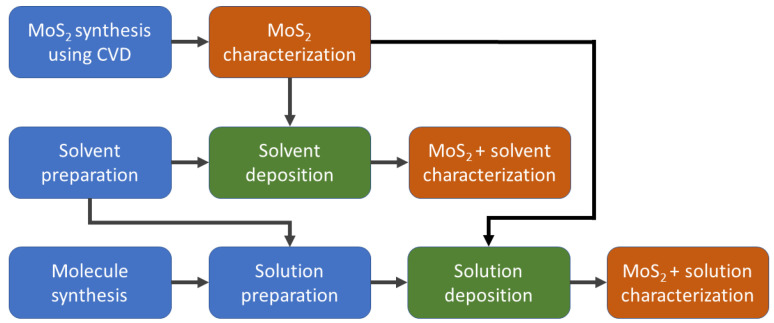
Flow chart depicting the order of sample preparation and measurements. The chart shows the steps taken to measure sample properties after synthesis, solvent drop-casting and organic molecule solution drop-casting. The chart begins with the synthesis step and proceeds to the measurement of sample properties at various stages, as indicated by the arrows.

For distinguishing and comparing the impacts of various stages, the MoS${}_{2}$ islands were analyzed post-synthesis, subsequent to the solvent’s drop-casting and following the organic molecule solution’s drop-casting (Figure 2).

For the solvent and solution drop-casting, a 2.5 μL drop of selected solvent was placed on the sample and allowed to evaporate under ambient conditions.

## 3. Results and Discussion

### 3.1. Influence of Solvents on MoS${}_{2}$

In this section, we present a comprehensive investigation into the effects of various solvents on the properties of MoS${}_{2}$. The primary objective of this study was to identify an optimal solvent for droplet modification of MoS${}_{2}$ samples without causing detrimental effects on the material’s structural, morphological and optoelectronic properties.

Choosing an appropriate solvent is crucial for ensuring the effectiveness of the proposed method, as it can significantly influence the interactions between the solvent molecules, the functional groups and the target material [46,47,48]. A suitable solvent should exhibit minimal interaction with the MoS${}_{2}$ surface, ensuring that the material’s intrinsic properties remain unaffected during the process. The solvent should completely wet the substrate in order to uniformly cover the surface with organic molecules and avoid larger aggregations. Additionally, the solvent should evaporate completely without leaving any residue or traces that could affect the properties of MoS${}_{2}$. This minimizes the risk of introducing defects, impurities or unwanted modifications into the material, thereby preserving the integrity and quality of the 2D MoS${}_{2}$ samples.

In our experiments, we investigated the impact of a variety of solvents, including water, acetone, methanol, ethanol and isopropanol, on the properties of MoS${}_{2}$. These solvents were selected based on their widespread use, accessibility and varying physicochemical properties.

The subsequent sections present a detailed discussion on the outcomes observed for each solvent and their implications for the droplet modification of MoS${}_{2}$ samples. Additionally, the insights gained from this study may also contribute to a broader understanding of solvent–material interactions in the context of 2D materials and their modification.

#### 3.1.1. Water and Acetone as Solvents for Droplet Modification: Unsuitable Outcomes

In the pursuit of finding suitable solvents of MoS${}_{2}$/SiO${}_{2}$ samples, water (Mili-Q, type 1) and acetone (99.99%, HPLC grade) were initially considered due to their low cost and availability. However, the results of using these solvents proved to be unfavorable, as they caused severe damage to the MoS${}_{2}$ samples. This damage rendered the samples unusable for further experiments and analysis, making water and acetone unsuitable choices for this method of modification.

Following the procedure described in Section 2.2, MoS${}_{2}$/SiO${}_{2}$ samples were subjected to droplet modification using water and acetone. During this process, a droplet of each solvent was placed on the 2D material, allowing the solvent molecules to interact with the material’s surface, and droplets were placed for the solvent evaporation at ambient conditions. Afterward, the solvent evaporates at ambient conditions; the 2D material surface quality is assessed. However, in the case of water and acetone, this process consistently led to severe damage and contamination of MoS${}_{2}$ sample surfaces with trace amounts of errant chemicals.

**Figure 3:**
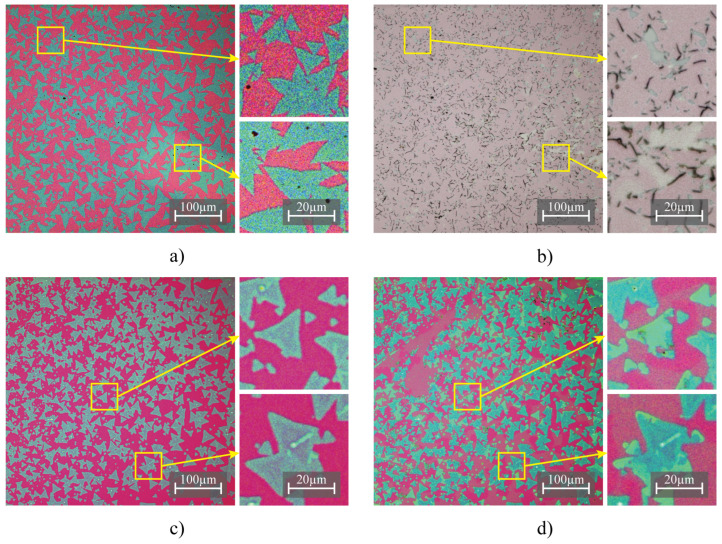
Optical microscopy images of MoS${}_{2}$ samples subjected to different solvent treatments, highlighting the morphology changes. Before water treatment (**a**), after water treatment (**b**), before acetone treatment (**c**) and after acetone treatment (**d**). Note that changes in color and noise are due to automatic exposure set by the microscope.

Optical microscopy images provided clear evidence of the damaging effects of water and acetone on MoS${}_{2}$ samples. Figure 3 displays optical microscopy images of the samples before and after the solvent drop-casting.

Samples exposed to type 1 water exhibit detachment and folding of MoS${}_{2}$ monolayers with major defect formations. Water exhibited extremely poor wetting of the sample since the SiO${}_{2}$ substrate is well known to be hydrophobic. The large contact angle between water and the substrate induces strong out-of-plane force on the MoS${}_{2}$ during drying, causing it to slightly delaminate from the substrate. A common method to increase the wetting of water to amorphous SiO${}_{2}$ is by treating the SiO${}_{2}$ with oxygen plasma. However, our tests have shown that even a slight plasma treatment damages and oxidizes MoS${}_{2}$ samples on the substrate.

Although the droplet contact angle with the sample was relatively low while wetting was good, acetone still caused significant structural damage along with the formation of large cracks which split the monolayer into smaller pieces.

The strong interactions between the solvent molecules and the MoS${}_{2}$ layers, coupled with the stress generated during solvent evaporation, compromised the integrity of the samples. As a result, water and acetone, when used individually, cannot be considered viable solvents for this method of modification and should be noted as not suitable for use with MoS${}_{2}$. However, further investigations may explore their potential use in combination with other solvents or strategies to achieve the desired modification goals while minimizing damage to the MoS${}_{2}$ samples.

#### 3.1.2. Methanol and Ethanol as Solvents for Droplet Modification: Challenges and Limitations

Following the unfavorable outcomes observed with water and acetone, ethanol and methanol were investigated as alternative solvents for droplet modification of MoS${}_{2}$/SiO${}_{2}$ samples. As polar organic solvents, ethanol and methanol possess certain advantageous properties, such as low cost, low viscosity and high volatility, which make them potentially suitable candidates for this purpose. However, their use can lead to some undesirable effects on the sample quality, particularly in terms of stability and morphology.

**Figure 4:**
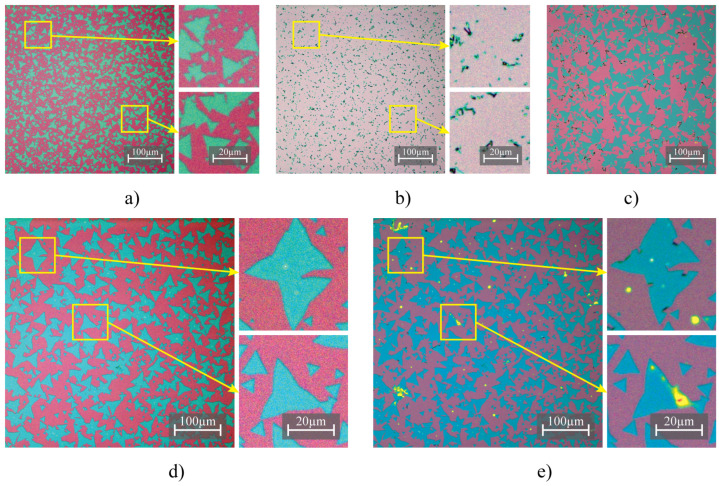
Optical microscopy images of MoS${}_{2}$ samples subjected to different solvent treatments, highlighting the morphology changes. Before ethanol treatment (**a**), disturbed area after ethanol treatment exhibiting folded and cracked MoS${}_{2}$ islands (**b**), second area on the sample after ethanol treatment with minor morphological changes (**c**), before methanol treatment (**d**) and after methanol treatment (**e**).

The application of ethanol and methanol in droplet modification followed the same procedure as with water and acetone, after which the solvent evaporated. However, this process was not reproducible and in some cases may lead to the formation of defects such as folding, cracks and other irregularities in the MoS${}_{2}$ samples, similar to water (Figure 4). Although in some cases, samples seem to mechanically survive the treatment, this was not reliably reproducible, making a lot of samples unsuitable for further characterization.

In contrast with the effects observed in the use of water and acetone, where the entirety of the area beneath the droplet underwent severe impairment, treatments involving ethanol and methanol resulted in less comprehensive damage and notable alterations. This relatively limited extent of impact potentially renders these latter substances more beneficial for modification processes. However, their inherent unpredictability and lack of reproducibility render them suboptimal for implementation in this particular methodology.

Therefore, we conclude that the use of methanol and ethanol as solvents for droplet modification of MoS${}_{2}$/SiO${}_{2}$ samples presents certain challenges and limitations. Their application in droplet modification can result in undesirable effects on the sample quality, particularly in terms of stability and morphology. Despite these drawbacks, methanol and ethanol may still hold potential for use in combination with other solvents or strategies that could mitigate the adverse effects and achieve the desired modification goals while preserving the quality of the MoS${}_{2}$ samples.

#### 3.1.3. Isopropanol as a Solvent for Droplet Modification: Advantages and Success

After exploring the use of water, acetone, ethanol and methanol as solvents for droplet modification of MoS${}_{2}$/SiO${}_{2}$ samples, isopropanol emerged as the most suitable solvent for this purpose. Isopropanol offers several advantages over the previously studied solvents, including repeatability, ease of handling and favorable viscosity. These properties contributed to the successful droplet modification of MoS${}_{2}$ samples, making isopropanol the preferred choice.

Isopropanol’s low viscosity and surface tension allows it to spread evenly over the MoS${}_{2}$ samples, ensuring uniform modification across a larger surface area. Moreover, it evaporates quickly, enabling rapid drying of the functionalized samples, which is essential for minimizing the formation of defects such as cracks and folds. An added benefit of using isopropanol is its ability to wash away any residual surface particles from the synthesis process to the edge of the droplet, further improving the quality and cleanliness of the MoS${}_{2}$ samples.

**Figure 5:**
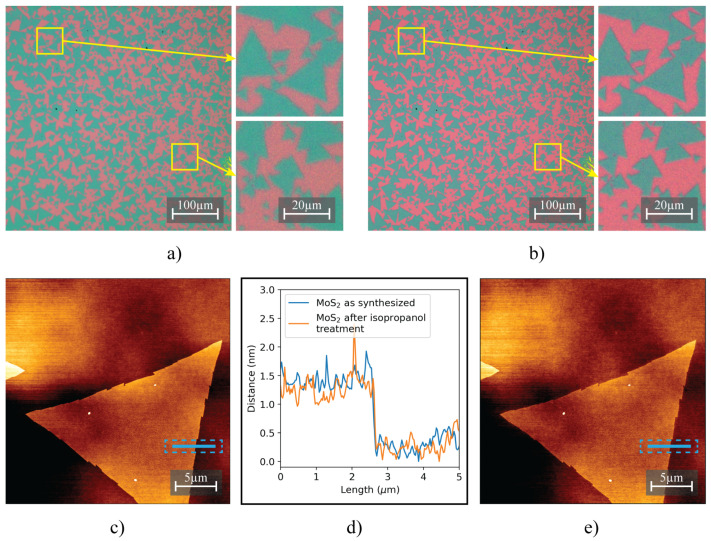
Optical microscopy and AFM images capturing the morphological measurements of MoS${}_{2}$ samples post-isopropanol treatment. Optical image before isopropanol drop-casting (**a**), optical image after the said treatment (**b**), AFM images before (**c**) and after isopropanol drop-casting (**e**) with line scans (indicated by blue lines with dashed outlines on both AFM images) that compare surface morphology before and after treatment shown in (**d**).

Optical microscopy and AFM images in Figure 5 demonstrate the successful application of isopropanol for droplet modification of MoS${}_{2}$/SiO${}_{2}$ samples.

The images show minimal change of the samples, with no visible cracks, folds or other irregularities, indicating that isopropanol is a reliable solvent to use for modification. The AFM image (Figure 5e) reveals a smooth surface morphology, confirming the absence of cracks and folds in the samples.

**Figure 6:**
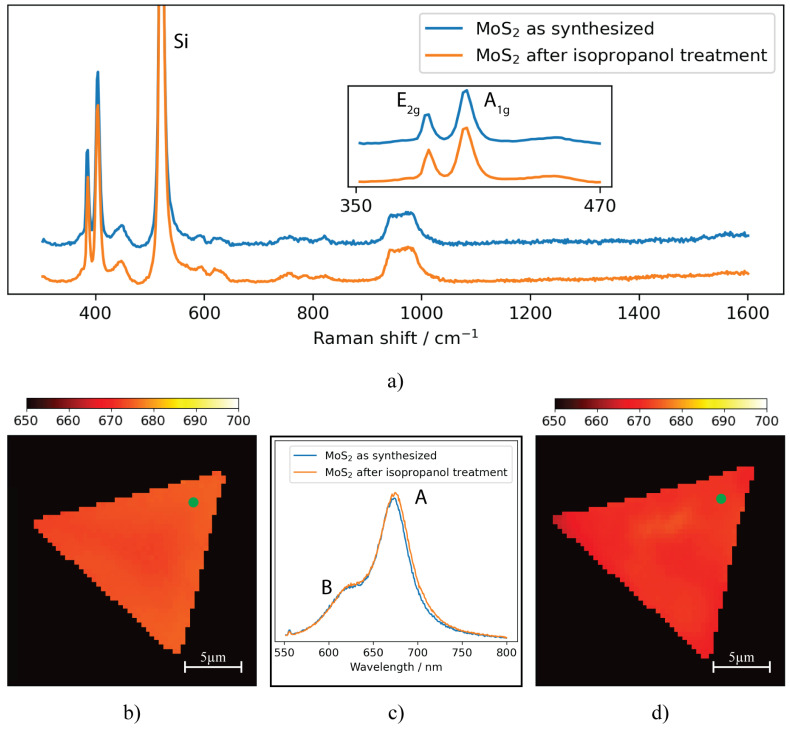
Measured Raman spectra (**a**), PL map of unmodified (**b**) and isopropanol treated MoS${}_{2}$ (**d**); and PL spectra (**c**) of MoS${}_{2}$/SiO${}_{2}$ samples before and after isopropanol treatment. Note: The green circle indicates the point used for individual plots and the PL map denotes the wavelength of the Exciton A peak.

Further characterization of the MoS${}_{2}$ samples treated with isopropanol was performed using Raman spectroscopy and PL measurements. The Raman spectra (Figure 6a) show characteristic MoS${}_{2}$ peaks, indicating that the material’s crystalline quality is preserved after isopropanol treatment. Ultimately, distinct emission peaks are observed in the PL spectra (as shown in Figure 6b–d), which is indicative of the preserved optoelectronic properties of the MoS${}_{2}$ samples. Notably, a small average exciton A shift of the exciton A band is detected from 671.9±0.7 nm to 673.8±0.8 nm. This small shift could be attributed to band gap narrowing due to strain release [49].

In conclusion, isopropanol has proven to be the optimal solvent for droplet modification of MoS${}_{2}$/SiO${}_{2}$ samples, outperforming water, acetone, ethanol and methanol. It repeatably induces negligible mechanical and chemical changes in the base material. It exhibits favorable properties, such as ease of handling and suitable viscosity and thus is able to achieve uniform modification across a larger surface area while minimizing defects. Additionally, isopropanol’s quick evaporation and ability to remove residual contaminants further improve the cleanliness of the MoS${}_{2}$ samples. The AFM, Raman and PL characterization results confirm the preservation of the material’s structural, crystalline and optoelectronic properties after the treatment with isopropanol.

### 3.2. Modification of MoS${}_{2}$ Samples Using L63MS Molecule

Building on our encouraging findings using isopropanol for droplet modification of MoS${}_{2}$/SiO${}_{2}$ samples, we proceeded to integrate the L63MS molecule with isopropanol to influence the properties of MoS${}_{2}$. We introduce L63MS, a complex but water-soluble molecule, as part of our evolving approach to evaluate the impact of solvent selection. Despite its insolubility in isopropanol, we chose L63MS due to its clear alteration of material properties, reinforcing the efficacy of our method while also demonstrating that isopropanol-soluble molecules are not the only option. Given the solubility profile of the L63MS molecule—soluble in water but insoluble in isopropanol—we utilized a mixed solvent system. The L63MS molecule was first dissolved in water to the concentration of $10^{-2}$ M as a stock solution. The stock solution was further dissolved in pure isopropanol, resulting in a concentration of L63MS molecule of $10^{-4}$ M and 99.5% *v*/*v* isopropanol solution. We employed this final solution, containing a L63MS concentration of $10^{-4}$ M, for the droplet modification of MoS${}_{2}$ samples.

For this investigation, we focused exclusively on MoS${}_{2}$ samples functionalized with the L63MS in the 99.5% isopropanol and 0.5% water solution. Notably, our initial experiments indicated no significant alterations in morphological and optical properties between using pure isopropanol and the 99.5% isopropanol with 0.5% water solution, thus validating our choice of the mixed solvent for this study.

**Figure 7:**
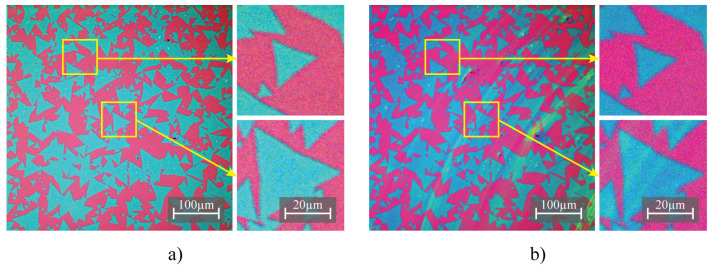
Optical microscopy images of MoS${}_{2}$/SiO${}_{2}$ samples at different stages of treatment. MoS${}_{2}$ after synthesis (**a**), showcasing the initial morphology and MoS${}_{2}$ after L63MS solution drop-casting (**b**). The surface color variations observed via optical microscope, from a lighter shade of blue to a subtle shift towards green, represent changes in the molecule concentration. The shift towards green, although slight, indicates regions of higher molecule concentration.

Optical microscopy images of the MoS${}_{2}$ samples before and after modification are shown in Figure 7.

**Figure 8:**
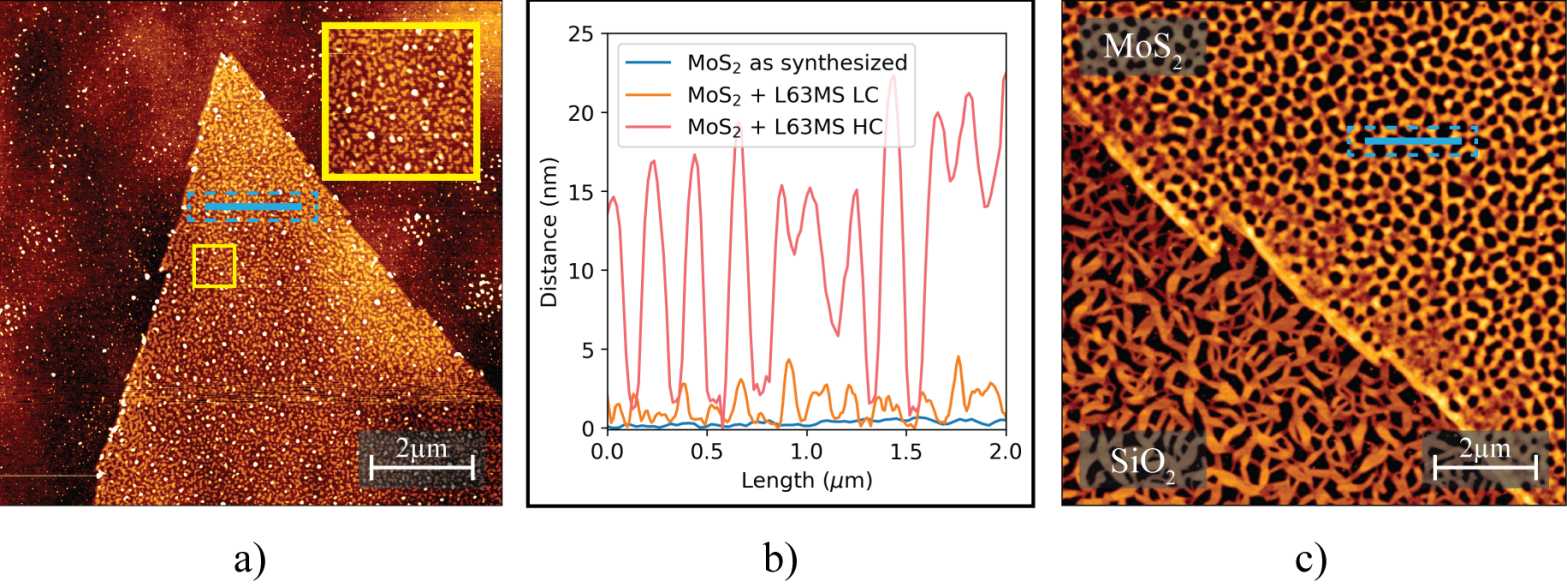
AFM images of MoS${}_{2}$/SiO${}_{2}$ samples treated with L63MS solution at different positions on the sample. Image of an area with a lower amount (LC) of molecules after deposition (**a**), an area with a higher amount (HC) of molecules (**c**) and a cross-sectional comparison (line scans are indicated by blue lines with dashed outlines on both AFM images) of each image illustrating the height profile of the molecules at the respective positions (**b**).

Significantly, the images demonstrate that the modification process, utilizing the L63MS in an isopropanol–water solution, maintains the material quality by not inducing detectable damage or altering the sample morphology. Although the cast droplet wets the sample uniformly, the concentration of L63MS after drying exhibits variations across the surface as seen on Figure 7b. To further investigate this uneven molecular deposition, AFM is employed to analyze two regions characterized by lower and higher molecular density. Figure 8 presents AFM images of two distinct MoS${}_{2}$ samples after post-modification.

The captured images display that a noticeable quantity of L63MS molecules adhered to the sample surface. Despite this, the surface retains its integrity without the presence of cracks or folds. The variance in molecular coverage, dependent on the relative positions to the droplet location, elucidates the inhomogeneous nature of the drop-casting process. Note that the thickness of the molecular layer in the lower concentration MoS${}_{2}$ section varies from 0.5 to 4 nm, in contrast to the 5 to 20 nm range found on the higher concentration MoS${}_{2}$ section. The molecular layer thickness on the lower concentration MoS${}_{2}$ concurs with the dimensions expected of a monolayer of molecules, although this can be orientation-dependent. In the interest of this study, subsequent analysis will focus on the lower concentration sample to elucidate the implications of a singular molecular layer.

**Figure 9:**
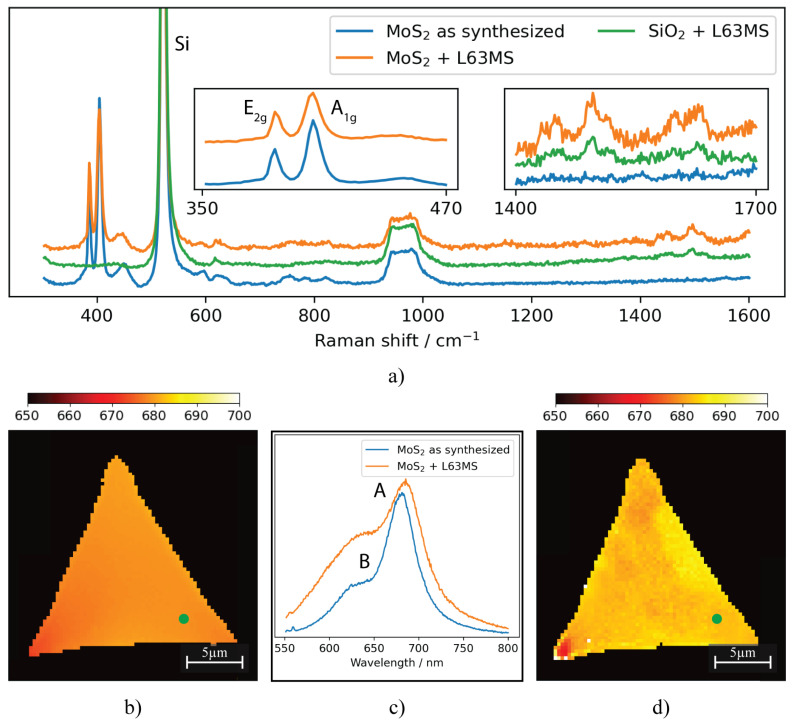
Raman graph (**a**), PL map before drop-casting (**b**) and after modification with L63MS (**d**), and an example PL spectra (**c**) for both cases. Note: The green circle indicates the point used for individual plots and the PL map denotes the wavelength of the Exciton A peak.

Raman spectra for the lower concentration sample before and after drop-casting are displayed in Figure 9a.

The distinctive peaks characteristic of MoS${}_{2}$ are discernible in all samples, indicating that the crystalline quality remains intact throughout the modification process. However, additional unique peaks between 1400 and 1700 nm not found in clean or isopropanol–water treated samples become detectable. Similar characteristic peaks can be identified when performing Raman spectroscopy on the molecules present on a clean SiO${}_{2}$ sample. A meticulous analysis of these Raman spectra could uncover minor alterations in the MoS${}_{2}$ properties, which would signal a stronger binding of the L63MS molecule.

PL spectra for the lower concentration sample before and after drop-casting are shown in Figure 9b–d. The two emission peaks (named A and B excitons) are stemming from the two exciton states, which are optical transitions at the K point from two spin-split bands of the reciprocal lattice [50,51]. The distinct emission peaks observed in the spectra suggest that while there are some alterations, the optoelectronic properties of the MoS${}_{2}$ samples are maintained after modification. Notably, our observation post-modification shows a pronounced shift in the exciton A peak from 680±1 nm to 688±1 nm and a modification of the A/B exciton peak ratios. This shift is markedly greater than what we observed from only isopropanol and isopropanol–water covered MoS${}_{2}$ islands, implying a change in the material (opto)electronic properties. Considering these observations, we hypothesize that these alterations could be the direct result of MoS${}_{2}$ doping, which impacts the excitonic structure and hence modifies the peak positions and relative intensities [52,53,54]. We intend to further explore these changes in our future studies, focusing particularly on understanding the physics driving these spectral shifts.

To summarize, the modification of MoS${}_{2}$ samples using the L63MS molecule in combination with the water–isopropanol mixed solvent system has been demonstrated to be a viable route toward organic modification of MoS${}_{2}$ under ambient pressure conditions. The characterization results from optical microscopy, AFM and Raman spectroscopy indicate that the overall quality, crystallinity and surface morphology of the MoS${}_{2}$ samples are preserved after modification. PL measurements demonstrate a slight alteration in optical properties that could be attributed to doping. This study highlights the potential of using L63MS and isopropanol to modify the properties of MoS${}_{2}$ and provides a foundation for further investigation the modification of other 2D materials using similar approaches.

## 4. Conclusions

In conclusion, our study offers crucial insights into how different solvents impact 2D MoS${}_{2}$ and the potential to modify it with organic molecules. Our findings suggest that water and acetone negatively affect MoS${}_{2}$’s structure and morphology, while ethanol and methanol show potential but face stability and reproducibility issues. Isopropanol stands out as the best solvent for drop-casting organic solutions on MoS${}_{2}$, maintaining its properties without significant defects. Alongside the L63MS-water solvent system, this proves promising for modifying 2D materials.

Future research will involve studying the long-term impact of deposited molecules on stability, conductivity, optical response and thermal characteristics. We also plan to examine the effects of drop-casting parameters, like temperature, pressure and time, on the modification process.

## Data Availability

The data presented in this study are available on request from the corresponding author. The data are not publicly available due to their involvement in ongoing research and the fact that all pertinent information for the comprehension of this specific study is comprehensively presented within the paper itself.

## Abbreviations

The following abbreviations are used in this manuscript:

2D	Two-dimensional
L63MS	6-(4,5-Dihydro-1*H*-imidazol-3-ium-2-yl)-2-(naphthalene-2-yl)benzothiazole methanesulfonate
AFM	Atomic Force Microscopy
BP	Black Phosphorus
CVD	Chemical Vapor Deposition
hBN	Hexagonal Boron Nitride
MoS${}_{2}$	Molybdenum Disulfide
PL	Photoluminescence
SiO${}_{2}$	Silicon Dioxide
TMDC	Transition Metal Dichalcogenide
vdW	Van der Waals
